# Muscle Cell Morphogenesis, Structure, Development and Differentiation Processes Are Significantly Regulated during Human Ovarian Granulosa Cells In Vitro Cultivation

**DOI:** 10.3390/jcm9062006

**Published:** 2020-06-26

**Authors:** Claudia Dompe, Wiesława Kranc, Karol Jopek, Katarzyna Kowalska, Sylwia Ciesiółka, Błażej Chermuła, Artur Bryja, Maurycy Jankowski, Joanna Perek, Małgorzata Józkowiak, Lisa Moncrieff, Greg Hutchings, Krzysztof Janowicz, Leszek Pawelczyk, Małgorzata Bruska, James Petitte, Paul Mozdziak, Magdalena Kulus, Hanna Piotrowska-Kempisty, Robert Z. Spaczyński, Michał Nowicki, Bartosz Kempisty

**Affiliations:** 1 The School of Medicine, Medical Sciences and Nutrition, University of Aberdeen, Aberdeen AB25 2ZD, UK; claudia.dompe.16@abdn.ac.uk (C.D.); l.moncrieff.16@abdn.ac.uk (L.M.); g.hutchings.16@abdn.ac.uk (G.H.); krzysztof.janowicz.16@abdn.ac.uk (K.J.); 2 Department of Histology and Embryology, Poznan University of Medical Sciences, 6 Święcickiego St., 60-781 Poznan, Poland; karoljopek01@gmail.com (K.J.); kkowalsk@ump.edu.pl (K.K.); sciesiolka@ump.edu.pl (S.C.); mnowicki@ump.edu.pl (M.N.); 3 Department of Anatomy, Poznan University of Medical Sciences, 6 Święcickiego St., 60-781 Poznan, Poland; wkranc@ump.edu.pl (W.K.); abryja@ump.edu.pl (A.B.); mjankowski@ump.edu.pl (M.J.); joanna.per2000@gmail.com (J.P.); mbruska@ump.edu.pl (M.B.); 4 Division of Infertility and Reproductive Endocrinology, Department of Gynecology, Obstetrics and Gynecological Oncology, Poznan University of Medical Sciences, 33 Polna St., 60-535 Poznan, Poland; blazej.chermula@wp.pl (B.C.); pawelczyk.leszek@ump.edu.pl (L.P.); rspaczynski@yahoo.com (R.Z.S.); 5 Department of Toxicology, Poznan University of Medical Sciences, 30 Dojazd St., 60-631 Poznan, Poland; malgorzata.jozkowiak@gmail.com (M.J.); hpiotrow@ump.edu.pl (H.P.-K.); 6 Prestage Department of Poultry Science, North Carolina State University, Raleigh, NC 27695, USA; jnppo@ncsu.edu; 7 Physiology Graduate Program, North Carolina State University, Raleigh, NC 27695, USA; pemozdzi@ncsu.edu; 8 Department of Veterinary Surgery, Institute of Veterinary Medicine, Nicolaus Copernicus University in Torun, 1 Lwowska St., 87-100 Toruń, Poland; magdalena.kulus@umk.pl; 9 Department of Obstetrics and Gynecology, University Hospital and Masaryk University, 20 Jihlavská St., 62500 Brno, Czech Republic

**Keywords:** human GCs, in vitro culture, proliferation, muscle differentiation

## Abstract

Granulosa cells (GCs) have many functions and are fundamental for both folliculogenesis and oogenesis, releasing hormones and communicating directly with the oocyte. Long-term in vitro cultures of GCs show significant stem-like characteristics. In the current study, RNA of human ovarian granulosa cells was collected at 1, 7, 15 and 30 days of long-term in vitro culture. Understanding the process of differentiation of GCs towards different cell lineages, as well as the molecular pathways underlying these mechanisms, is fundamental to revealing other possible stemness markers of this type of cell. Identifying new markers of GC plasticity may help to understand the aetiology and recurrence of a wide variety of diseases and health conditions and reveal possible clinical applications of the ovarian tissue cells, affecting not only the reproductive ability but also sex hormone production. Granulosa cells were the subject of this study, as they are readily available as remnant material leftover after in vitro fertilisation procedures and exhibit significant stem-like characteristics in culture. The change in gene expression was investigated through a range of molecular and bioinformatic analyses. Expression microarrays were used, allowing the identification of groups of genes typical of specific cellular pathways. This candidate gene study focused on ontological groups associated with muscle cell morphogenesis, structure, development and differentiation, namely, “muscle cell development”, “muscle cell differentiation”, “muscle contraction”, “muscle organ development”, “muscle organ morphogenesis”, “muscle structure development”, “muscle system process” and “muscle tissue development”. The results showed that the 10 most upregulated genes were keratin 19, oxytocin receptor, connective tissue growth factor, nexilin, myosin light chain kinase, cysteine and glycine-rich protein 3, caveolin 1, actin, activating transcription factor 3 and tropomyosin, while the 10 most downregulated consisted of epiregulin, prostaglandin-endoperoxide synthase 2, transforming growth factor, interleukin, collagen, 5-hydroxytryptmine, interleukin 4, phosphodiesterase, wingless-type MMTV integration site family and SRY-box 9. Moreover, ultrastructural observations showing heterogeneity of granulosa cell population are presented in the study. At least two morphologically different subpopulations were identified: large, light coloured and small, darker cells. The expression of genes belonging to the mentioned ontological groups suggest the potential ability of GCs to differentiate and proliferate toward muscle lineage, showing possible application in muscle regeneration and the treatment of different diseases.

## 1. Introduction

Together with oocytes, theca cells and ovarian surface epithelial cells, granulosa cells (GCs) are found in the follicular fluid and are fundamental for folliculogenesis and oogenesis [1,2,3,4]. GCs are involved in the communication among cells, for example, releasing exosomes, small vesicle secreting microRNAs, and forming gap junctions [5,6]. Mural GCs surround the oocyte, forming the follicular wall around the antrum and communicate with the female gamete through the cumulus cells, which remain in direct contact with the oocyte and, through gap junctions, allow bi-directional stimulation as well as reciprocal exchange of nutrients and metabolites promoting folliculogenesis and oogenesis [7]. In fact, GCs stimulate the maturation of oocytes which, in turn, promote GCs proliferation and differentiation required for proper follicular growth. Moreover, they display a structural and functional role in the active preovulatory and ovulatory follicle.

The different functions played by GCs in the endocrine system are observed to change during long-term culture. In fact, GCs can differentiate towards multiple different cell lineages showing great potential for diverse clinical therapies and applications. Kossowska-Tomaszczuk et al. showed that GCs have high proliferation capability and differentiational potential towards lineages such as neurons, osteoblasts and chondrocytes [8,9]. Further studies revealed the ability of GCs to differentiate into muscle cells and cardiac cells [10,11].

Understanding the process of differentiation of GCs towards different cell lineages, as well as the molecular pathways underlying these mechanisms is fundamental to reveal other possible stemness markers of this type of cells. Modifications of cell culture, such as 3D cultures, MEF (mouse embryonic fibroblast) medium, follicular fluid and leukaemia inhibitory factor, have been reported to prolong the lifespan and stemness of GCs as well as enhance proliferation and ability to maintain cells in an undifferentiated state [8,12,13]. These techniques may be useful to fully take advantage of the stem-like potential of GCs. In fact, under these specific culture conditions, GCs show changes in stem cell phenotype, for example expressing genes characteristic for heart morphogenesis [10]. GCs also exhibited osteogenic differentiation potential under long-term in vitro culture conditions [14]. The present research describes the transcriptomic profile of human GCs during their long-term primary culture and, through candidate gene approach, aims to identify new markers of GC plasticity through the expression of genes belonging to ontology groups associated with muscle cells morphogenesis, structure, development and differentiation. Additionally, it explores the ultrastructural alterations in the cultured GCs, relating the findings to the possible changes of their morphology and phenotype.

## 2. Experimental Section

### 2.1. Patients Characteristics

Human follicular fluid (FF) with suspended GCs was obtained from 20 infertile patients (mean age 33.67 years ± 1.46 (SEM; standard error of the mean), range 25–40) undergoing IVF-ICSI procedure at the Centre of Diagnosis and Treatment of Infertility in the Division of Infertility and Reproductive Endocrinology at the Poznan University of Medical Sciences. Controlled ovarian hyperstimulation was induced by the administration of recombinant human follicle-stimulating hormone (rhFSH; Gonal F, Merck sp. z o.o, Poland or Puregon, MSD Poland sp. z o.o, Poland) and highly purified human menopausal gonadotropin (hMG; Menopur, Ferring Pharmaceuticals Poland sp. z o.o, Poland) in individualized doses, according to the designed protocol (Cetrotide, cetrorelix 0.25 mg, Merck sp. z o.o, Poland or Orgalutran, ganirelix 0.25 mg, MSD Poland sp. z o.o, Poland). Ovulation was induced with rh chorionic gonadotropin (rhCG; Ovitrelle, 250 μg, Merck sp. z o.o, Warszawa, Poland). FF was obtained by transvaginal ultrasound-guided aspiration, 36 h after rhCG administration. The selected infertile female patients had no history of ovarian surgery, no other chronic or endocrine diseases, and a BMI < 30 kg/m^2^. Female patients with polycystic ovarian syndrome (PCOS), endometriosis, premature ovarian insufficiency (POI) and with poor ovarian response were excluded from the study.

Poznan University of Medical Sciences Bioethical Committee gave approval for the presented research (resolution nr 558/17). All patients qualified for the study, meeting the criteria for participation, gave informed written consent to participate in this study.

### 2.2. Long-Term In Vitro Culture of hGCs

In the first stage, the obtained FF was centrifugated at 200× *g* for 10 min at room temperature (RT). Next, the pellet with GCs was washed twice in culture medium and was centrifugated again with the same settings. The culture medium consisted of DMEM (Sigma; Merck KGaA, Darmstadt, Germany), 2% foetal bovine serum FBS (FBS; Sigma; Merck KGaA, Darmstadt, Germany), 10 mg/mL gentamicin (Invitrogen; Thermo Fisher Scientific, Inc., Waltham, MA, USA), 4 mm L-glutamine (stock 200 mm, Invitrogen; Thermo Fisher Scientific, Inc., Waltham, MA, USA), 10,000 μg/mL streptomycin and 10,000 U/mL penicillin (Invitrogen; Thermo Fisher Scientific, Inc., Waltham, MA, USA) [15,16,17].

All GCs cultures were incubated at 37 °C and 5% CO_2_. The GCs were cultured in flasks. When 90% confluency was reached, the cells were detached from the bottom using 0.05% trypsin–EDTA (Invitrogen; Thermo Fisher Scientific, Inc., Waltham, MA, USA) for 1–2 min and were counted with the ADAM Cell Counter and Viability Analyzer (Bulldog Bio, Portsmouth, NH, USA) (Adam CCVA). The long-term culture was carried out for 30 days. The culture medium was changed twice a week. ADAM CCVA was used to assess cell viability. Each sample was tested and samples containing 95% or more viable cells were used for further culture and molecular analysis [18,19].

### 2.3. RNA Extraction

Total RNA from cultured cells was extracted after 1, 7, 15, and 30 days of in vitro culture using the Chomczyński–Sacchi method [20]. Cells harvested at these specific timed intervals of culture were suspended in 1 mL of phenol and guanidine thiocyanate monophase solution (TRI Reagent^®^, Sigma; Merck KGaA, Darmstadt, Germany). Chloroform (0.2 mL per 1 mL TRI Reagent) was added to the prepared samples to obtain three separate phases. RNA was located in the topmost, aqueous phase. Then, the RNA was stripped with 2-propanol (Sigma; Merck KGaA, Darmstadt, Germany, catalogue number I9516) and washed with 75% ethanol. Extracted RNA from each sample was used for further molecular analysis. The total amount of mRNA was determined from the optical density at 260 nm, and the RNA purity was estimated using the 260/280 nm absorption ratio (NanoDrop spectrophotometer, Thermo Scientific, ALAB, Poland). Only samples with absorbance ratio 260/280 > 1.8 were used [16,21,22].

### 2.4. Microarray Expression Analysis

Before reverse transcription, the integrity of RNA samples was examined using gel electrophoresis, as requested in the microarray manufacturer protocol. Total RNA (100 ng) was converted to double-stranded cDNA. In the next step, labelled complementary RNA (cRNA) was synthesized and amplified by in vitro transcription of the double-stranded cDNA template (GeneChip^TM^ 3′IVT PLUS Reagent Kit, Applied Biosystems^TM^, Foster City, CA, USA). The obtained cRNA was fragmentated by divalent cations and elevated temperature. Fragmentated and labelled cRNA (7.5 μg) was hybridized to Human Genome U219 Array Strip (45 °C/16 h, Applied Biosystems^TM^, Foster City, CA, USA). Then, the microarrays were washed and stained according to the technical protocol using Affymetrix GeneAtlas Fluidics Station. Subsequently, the array strips were scanned by the Imaging Station of the GeneAtlas System. The preliminary analysis of the scanned chips was performed using the Affymetrix GeneAtlas^TM^ Operating Software. The quality of gene expression data was verified according to the quality control criteria provided by the software. The obtained CEL files were imported into a downstream data analysis software. All of the presented analyses and graphs were performed using Bioconductor and R programming language. Each CEL file was merged with a description file. To correct background, normalize and summarize the results, the Robust Multiarray Averaging (RMA) algorithm was used.

### 2.5. RT-qPCR Analysis

The validation of the results obtained during microarray analysis was performed using quantitative RT-qPCR. Twenty selected genes were validated, 10 of the most upregulated and 10 of the most downregulated. The validation was performed in three biological replicates. Each biological test was performed in three technical replications in order to detect repeatability and eliminate possible technical errors. Reverse transcription was performed according to the reagent protocol provided by the manufacturer—SABiosciences (RT2 First Stand kit-330401), using a Verlerimer 96 well thermocycler. For each reaction, 0.2 µg of RNA transcript was used.

Real-time PCR was performed using Light Cycler^®^ 96 (Roche Diagnostic GmbH, Germany); Master Mix RT2 SYBR^®^ Green ROX™ qPCR (Qiagen Sciences, Gaithersburg, MD, USA) and sequence-specific primers (Table 1). Gene expression was identified with the relative quantification (RQ) method, using 3-phosphate glyceraldehyde dehydrogenase (*GADPH*), β-actin (*ACTB*) and hypoxanthine 1 phosphoribosyltransferase (*HPRT1*) genes as reference. The RT-qPCR primers were designed using Primer3Plus software (primer3plus.com/cgi-bin/dev/primer3plus.cgi).

### 2.6. Electron Microscopy

Transmission electron microscopy analysis was conducted on the cell pellet containing a fraction of human ovarian granulosa cells, collected on the 1st, 7th, 15th and 30th day of long-term primary culture. Cells obtained from follicular fluid samples after 1 day culture were used as negative controls. The samples were fixed in buffered 2.5% glutaraldehyde solution. Next, they were washed three times in 7.2 pH phosphate buffer and finally fixed in 1% buffered OsO_4_ solution. After further triple washing in the same phosphate buffer, the cells were dehydrated in increasing concentrations of ethanol (50%, 70% 80%, 90%, 96% and 100%, respectively) at 4 °C. Furthermore, the cell pellet was placed in an ethanol/acetone solution, followed by acetone/resin mix. The cells were infiltrated in Epon-812 epoxy resin. Semi-thin and ultrathin slices were cut using a Leica Ultracut UCT ultramicrotome (Leica Microsystems, Nussloch, Germany). The initial evaluation of the representativity of the studied material was conducted, under a light microscope, on semi-thin slices (0.3 µm thick) stained with toluidine blue. Ultrathin slices (70 nm), placed on copper meshes (150 mesh), were stained with uranyl acetate and lead citrate. Then, they were subjected to analysis using a transmission electron microscope (TEM) model JEM 1010 (Jeol, Tokio, Japan).

### 2.7. Statistical Analysis

The statistical significance of the analysed genes was performed using moderated *t*-statistics from the empirical Bayes method. Obtained *p*-values were corrected for multiple comparisons using the Benjamini and Hochberg’s false discovery rate. The selection of significantly changed gene expression was based on a *p*-value beneath 0.05 and expression fold-change higher than 2. Differentially expressed genes were subjected to selection through their involvement in muscle cells morphogenesis, structure, development and differentiation. Differentially expressed gene list were uploaded to the DAVID software (Database for Annotation, Visualization and Integrated Discovery), where “muscle cell development”, “muscle cell differentiation”, “muscle contraction”, “muscle organ development”, “muscle organ morphogenesis”, “muscle structure development”, “muscle system process” and “muscle tissue development” GO BP terms were obtained. Expression data of these genes was subjected to hierarchical clustering procedure and presented as heatmap graphs. Detailed analysis of genes belonging to selected GO BP terms was presented as plots using the “GOplot” library [23].

Moreover, a list of differentially expressed genes from the selected GO BP terms was uploaded to the STRING software (Search Tool for Retrieval of Interacting Genes/Proteins) for interaction prediction.

Finally, the ReactomeFIViz app was used, it works with the Cytoscape software in order to create the Reactome Functional Interaction (FI) network from the set of differentially expressed genes.

The RT-qPCR results were also evaluated using the moderated *t*-statistics from the empirical Bayes method, corrected for multiple comparisons using Benjamini and Hochberg’s false discovery rate. The cut-off for statistical significance was assumed at *p* < 0.05.

## 3. Results

### 3.1. Microarray Results

**Figure 1:**
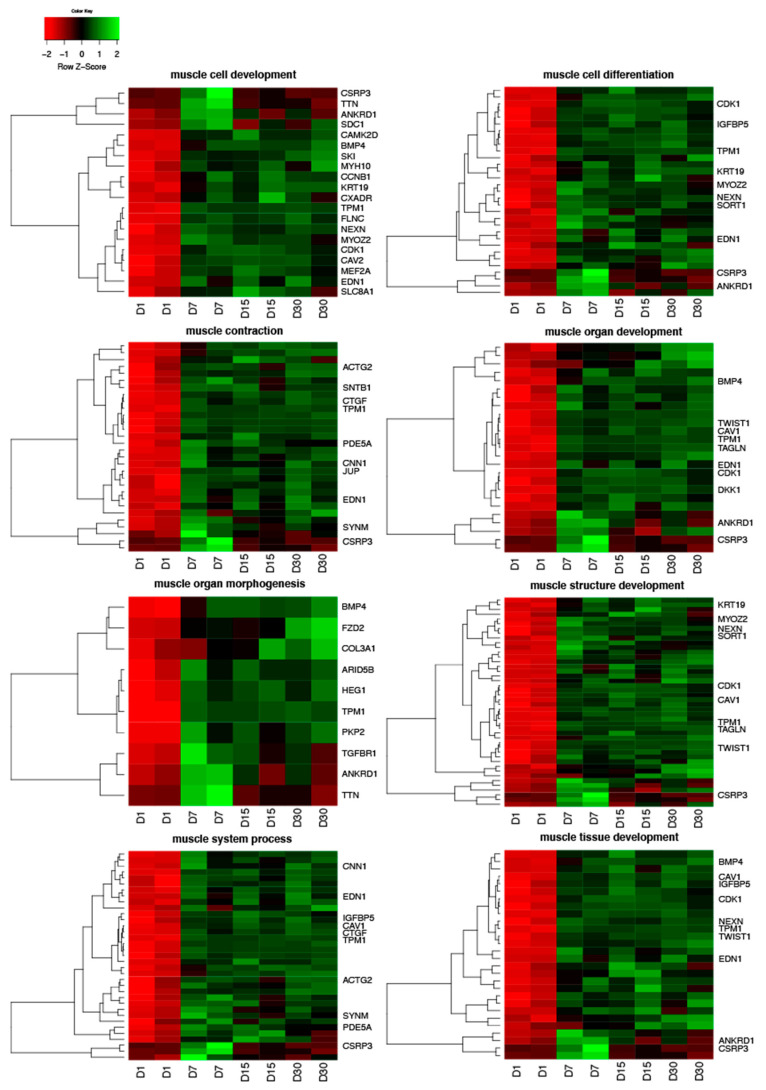
Heatmaps presenting differentially expressed genes involved in “muscle cell development”, “muscle cell differentiation”, “muscle contraction”, “muscle organ development”, “muscle organ morphogenesis”, “muscle structure development”, “muscle system process” and “muscle tissue development” based on GO BP (Gene Ontology Biological Process) terms. Each row on the *y*-axis represents a single transcript. The red colour indicates the downregulated genes while the green the upregulated genes.

Human Genome U219 Array Strips were used for the microarray gene expression analysis of human ovarian granulosa cells. This method allowed to study the gene expression of 22,480 transcripts at 1, 7, 15 and 30 days of in vitro granulosa cell culture. Genes selected for downstream analysis showed changes higher than 2-fold and corrected *p*-values less than 0.05. According to the above criteria, a total of 2579 differentially expressed genes (DEGs) were identified. The analysis of gene ontology families, enriched between DEGs, revealed 1989 GO terms. GO families that we focused on were ranked at positions between 153 and 387 according to number of represented genes and statistical significance, except for one GO term (“muscle organ morphogenesis”) which ranked at the 886th position. The DAVID software indicated the following GO BP terms, which cover the above processes: “muscle cell development”, “muscle cell differentiation”, “muscle contraction”, “muscle organ development”, “muscle organ morphogenesis”, “muscle structure development”, “muscle system process” and “muscle tissue development”. The 199 genes involved in those processes were hierarchically clustered and presented as heatmaps (Figure 1). One hundred and thirty-three genes were upregulated which is the greater part of the list of genes. The direction of expression change (upregulation or downregulation) in granulosa cell culture was maintained in subsequent points of analysis (7, 15, and 30 days of in vitro culture). The 10 most significantly upregulated and downregulated genes, their symbols, fold changes and corrected *p*-values are shown in Table 2.

**Figure 2:**
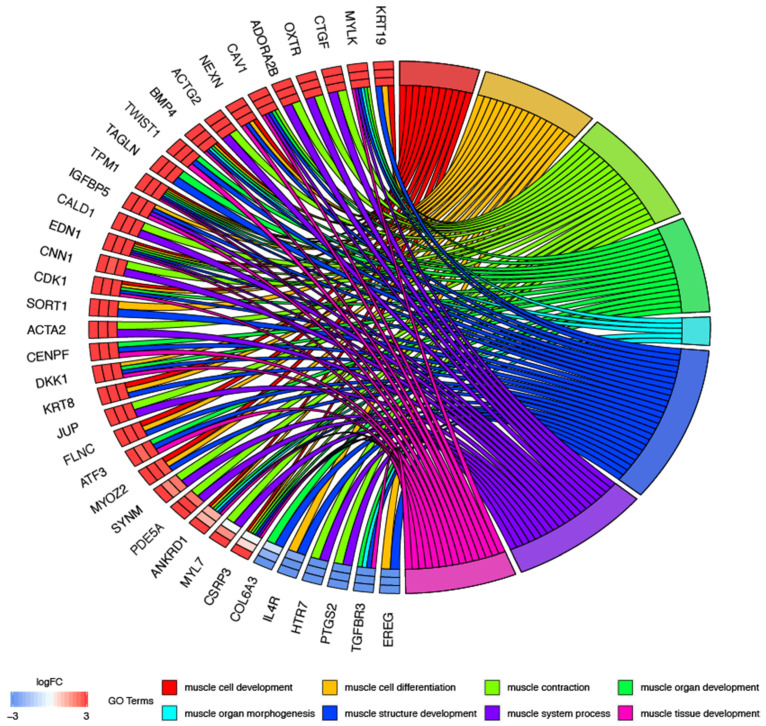
Analysis of enriched gene ontological groups involved in muscle cells morphogenesis, structure, development and differentiation. The network plot presenting the linkages of genes and GO BP terms.

In the next part of analysis, single genes may belong to many ontological terms. For this reason, the plots used had a visualization of the logFC values, and the relationship between genes and selected GO BP terms (Figure 2). The most upregulated genes from the examined GO BP terms included, among others, *KRT19*-keratin 19, *CTGF*-connective tissue growth factor, *OXTR*-oxytocin receptor and *NEXN*-nexilin. The strongest downregulated genes were *EREG*-epiregulin, *TGFBR3*-transforming growth factor, beta receptor III and *PTGS2*-prostaglandin-endoperoxide synthase 2.

**Figure 3:**
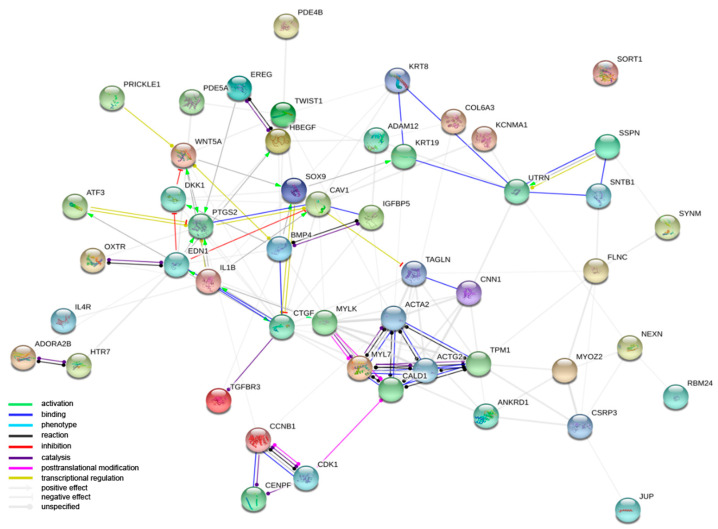
Interaction network of proteins encoded by the 50 most changed DEGs belonging to “muscle cell development”, “muscle cell differentiation”, “muscle contraction”, “muscle organ development”, “muscle organ morphogenesis”, “muscle structure development”, “muscle system process” and “muscle tissue development” GO BP terms. The network was generated by STRING software. Network nodes represent proteins. Empty nodes indicate proteins of unknown 3D structure.

The focus of the next analysis moved to the interactions among the proteins encoded by the DEGs belonging to the studied GO BP terms. Firstly, the STRING software was used for the interaction prediction. The number of genes used to create a STRING interaction network was limited, for readability, to the 50 most changed DEGs (Figure 3).

### 3.2. Validation of Gene Expression Direction

**Figure 4:**
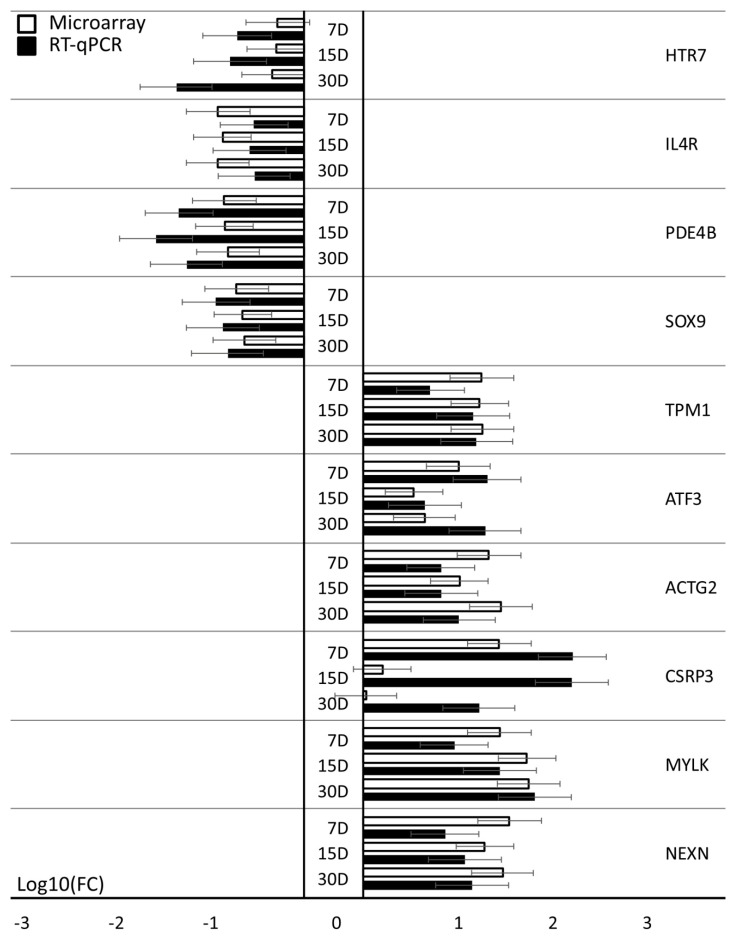
Microarray validation: RT-qPCR. The RT-qPCR results described validation of microarrays gene expression (log (FC)). Error bars represent the standard error mean (SEM; *n* = 3). All of the presented sample means were deemed to be statistically significant (*p* < 0.05). D: day of culture; FC: fold change.

The results obtained during the analysis of expression microarrays were validated using the RT-qPCR method. From the 10 most downregulated and 10 most upregulated genes, those that were not already examined in other works of our group (relating to other ontological groups, representing different cellular process or courses of potential differentiation) were subjected to validation, as they were exclusively indicated as markers for muscle development. The results are shown in Figure 4. Importantly, the direction of change in all 20 genes has been quantified. None of the genes showed any other change in expression than indicated by the results of the expression microarrays.

### 3.3. Ultrastructural Observation of Human Granulosa Cells

**Figure 5:**
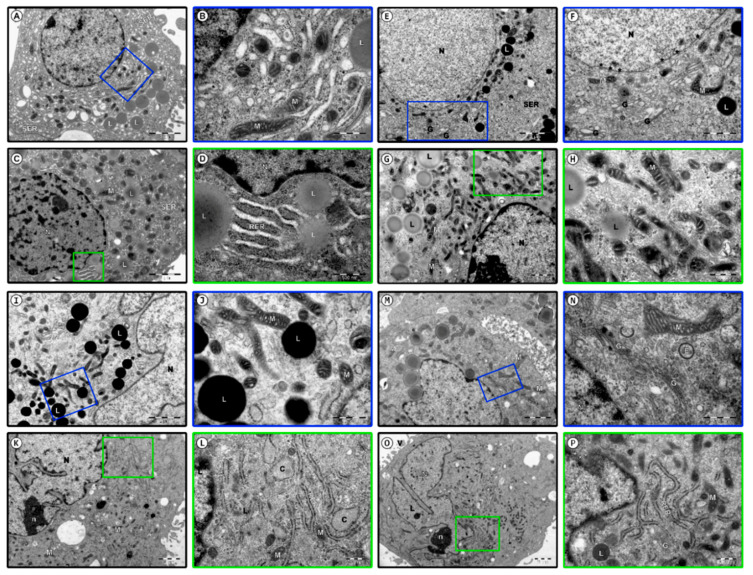
Photomicrographs of human ovarian granulosa cells during long-term culture. Day 1 (**A**–**D**); Day 7 (**E**–**H**); Day 15 (**I**–**L**); Day 30 (**M**–**P**); nucleus (**N**)**,** pores in the nuclear membrane (*), smooth endoplasmic reticulum (**SER**), mitochondria (**M**), lipid vacuoles (**L**), rough endoplasmic reticulum (**RER**), topical widenings (**C**), Golgi apparatus (**G**), endosomes (**E**), multivesicular bodies (**X**), microvilli (**V**), nucleolus (**N**).

Ultrastructural observations showed the heterogeneity of the granulosa cell population. At least two morphologically different subpopulations were identified, large and light coloured and small and dark cells. Follicular aspirate also contained cells of characteristics common to both sub-groups. The results of TEM observation were presented on Figure 5.

In the first day of the culture, cells from both populations exhibited properties of typical healthy, metabolically active steroidogenic cells. Those identified as large and bright (Figure 5A,B) were characterised by folded cell membrane of irregular shape. Large and eccentrically located euchromatin nucleus exhibited few peripheral chromatin foci. Multiple pores were detected in the nuclear membrane. Rich cytoplasm was filled with large network of smooth endoplasmic reticulum (SER) cisterns, characteristic for steroid cells, while rough endoplasmic reticulum (RER) cisterns were short and sparse. The cells had a well-developed Golgi apparatus, surrounded by numerous vesicles. Mitochondria, containing coil-like cristae, were localised in the space surrounding the nucleus. In the cytoplasm, numerous lipid vacuoles, as well as dispersed ribosomes and polysomes, can be observed.

Furthermore, on day 1 of culture, the cells identified as small and dark (Figure 5C,D) exhibited an electron-dense cytoplasm filled with numerous ribosomes, both free and aggregated, as well as polysomes. Folded cellular membrane contained numerous microvilli. Peripherally located irregularly shaped nucleus contained a number of dispersed, outwards located, heterochromatin foci. One or two prominent nucleoli were located inside the nucleus. The cytoplasm was filled with a dense network of SER cisterns and relatively numerous RER cisterns. A well-developed Golgi apparatus was surrounded by several vesicles of different shapes and sizes. In the cytoplasm, round or elongated pleomorphic mitochondria could be identified, exhibiting parallel or coil-like cristae. Numerous lipid vacuoles were present in all of the cells qualified to this group. Morphology of both sub-populations, as well as their cytoplasmic contents point to their high metabolic activity and protein synthesis ability.

In the 7th day of culture, the large, bright cells (Figure 5E,F) were characterised with rich cytoplasm with organelles placed near the nucleus. Round or elliptical nucleus with finely dispersed chromatin was surrounded by prominent envelope, with regularly distanced pores. A developed Golgi apparatus exhibited numerous, partly bloated, peripherally located membranous cisterns and vesicles. Numerous channels of the SER were observed, dispersed regularly in the cytoplasm. However, the diameter of its cisterns seemed to have decreased. Long, coil-like mitochondria with dark matrix were noted, as well as electron dense secretory granules, usually of lipid character. The small, dark cells (Figure 5G,H) exhibited nucleus with dispersed chromatin, condensed along the inner margin of the nuclear membrane. In the nucleus, a round, electron dense nucleolus was observed. Numerous elongated and diagonally located pleomorphic mitochondria were observed, mostly in the area surrounding the nucleus. The cytoplasm was filled with ribosomes, polysomes and microfilaments. A number of lipid vacuoles were also present.

In the 15th day of the culture, morphological changes were more noticeable. The volume of most of the large, bright cells (Figure 5I,J) increased compared to the 1st day of culture. Rich, bright cytoplasm seemed to be sparse. Enlarged, peripherally located nucleus assumed irregular outline. In the cytoplasm, numerous vesicular structures and peripherally located pinocytotic bodies were noted. The network of SER cisterns was scarcer.

The dark cells (Figure 5K,L) were characterised by large nuclei with numerous cavities, as well as electron dense irregular nucleolus. Most of the cells exhibited broad, branching RER cisterns, some showing topical widening. A decrease in number of lipid droplets was noted in this subpopulation.

In day 30 of cultures, cells of both subpopulations showed subtle morphological changes, associated with adaptation processes. Large, bright cells (Figure 5M,N) exhibited polarly located, irregularly shaped nucleus. Chromatin was usually regularly dispersed, with small peripheral foci of heterochromatin. The space surrounding the nucleus was filled with a developed Golgi apparatus, surrounded by numerous vesicles, endosomes and multivesicular bodies. In the Golgi cisterns, material of increased electron density could be observed. Pleomorphic mitochondria of heterogenous shapes were observed in the cytoplasm.

The volume of small, dark cells (Figure 5O,P) seemed to be notably bigger. The folded cellular membrane of irregular shape created numerous cytoplasmic protrusions and microvilli. Dark, rich cytoplasm contained large, polarly located polymorphic nucleus with a prominent nucleolus of large electron density. Pores could be noted in the nuclear envelope. Relatively rich RER showed numerous branches. Electron dense secretory granules were observed in some of the cells. Mitochondria present in the cytoplasm mainly exhibited regular cristae and electron dense matrix.

**Figure 6:**
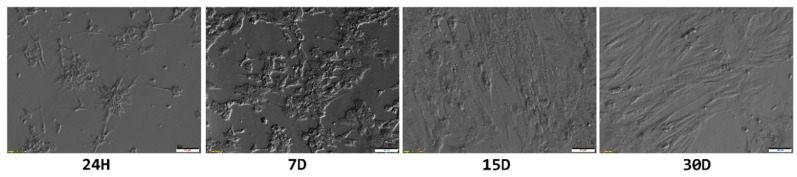
Light microscope observations of changes in granulosa cell morphology during long-term in vitro culture. H—hour; D—day.

These ultrastructural changes were also reflected in the overall morphology of cultured GCs (Figure 6). The shape of granulosa changed from small and star-like, to spindle-shaped, fibroblast-like, further supporting the notion of the change in their phenotype due to the lack of physiological conditions.

## 4. Discussion

Recent studies proved that most tissues and organs contain a population of stem cells with great differentiation potential, the ovary included [24,25,26]. Stem cells were found both in mouse and human ovaries and were observed to help oogenesis and folliculogenesis. Stem cells sustain the formation of oocyte and follicle in the postnatal mammalian ovary with some reports even suggesting that the consensus stating a defined number of primordial follicles formed in the embryonic period may in fact be wrong [27]. GCs were shown to possess great plasticity, showing stem cells properties, hence, great potential for regenerative medicine and transplantology. Furthermore, diverse publications demonstrate the ability of GCs to transdifferentiate, the process by which already differentiated cells can be reprogrammed towards a different cell lineage [28]. While some of the studies present a contrary view, debating if the presence of populations of stemness characteristics inside the ovary [29]. The reported properties of GCs observed during in vitro conditions are certainly worth investigating further.

As their functions in the reproductive system have been deeply studied and described, gene expression was hereby analysed through microarrays aiming to reveal the changes underlying the processes involved in the potential of GCs to differentiate towards muscle cells. Hence, using candidate gene approach, the current study identified and measured changes in expressions of genes belonging to specific ontological groups associated with this process of interest. The genes of most altered expression are presented below, shortly discussing their reported participation in both reproduction and muscle-associated processes.

KRT19 is a cytokeratin protein, the smallest of the KRTs family. Its expression is observed in endothelial cells and fibroblasts. Its expression may be useful for breast cancer detection, as it is observed in most epithelial tumour cells and was reported to play a key role in the stemness of hepatocellular carcinoma [30,31,32,33]. KRT19 was found to be the most upregulated and, as a cytoplasmic intermediate filament protein, it may be responsible for structural rigidity and scaffolding [34].

The second most expressed gene is responsible for the formation of the oxytocin receptor (oxytocin receptor—OXTR). Up to now, the expression of OXTR has been associated with many female reproductive pathways, such as myometrial and uterine contractility, control of the oestrous cycle length, cervical dilation and different pregnancy stages. OXTR is expressed in smooth muscle cells of tissues, such as the uterine myometrium and the mammary glands, and is responsible for contractions [35]. Recent research on mice models has suggested that OXTR is a key player in social behaviour [35]. OXTR was also found to have potentiality in skeletal muscle regeneration and differentiation as a paracrine agent. In fact, it is expressed in cultured human myoblasts and was shown to improve myoblast fusion and myotubule formation [36]. Moreover, Elabd et al. [37] have reported that OXTR is important for muscle cell regeneration and homeostasis.

Connective-tissue growth factor (CTGF) is involved in multiple cellular pathways, such as angiogenesis, osteogenesis and wound healing [38]. It regulates cell proliferation, differentiation, migration, apoptosis and extracellular matrix remodelling [39,40,41]. CTGF promotes growth and migration of vascular smooth muscle cells, its overexpression is observed in muscles of human muscular dystrophy, as well as induces apoptosis in human aortic smooth muscle cells [42,43,44].

NEXN (Nexilin F-actin binding protein), together with CSRP3 (cysteine-rich protein 3), also known as Muscle LIM protein (MLP), is shown to have important functions in foetal and adult hearts, skeletal muscle adhesion and migration during embryogenesis, and it is found to be involved in the development of cardiomyopathy [45,46]. Moreover, it is found to have a role in GLUT4 trafficking in skeletal muscles [47]. CSRP3 has an important role in muscle development, it is essential for myofiber differentiation and the architectural maintenance of muscle cells [48,49]. Tropomyosin (Tpm) is also important for skeletal muscle and the heart contractions [50,51]. In fact, it plays a role in the allosteric control of the actin filament of myosin which it can be inhibit or activate [52].

The gene coding for myosin light-chain kinase (MLCK) was also overexpressed. MLCK activates myosin motors affecting some contractile processes, such as the ones responsible for smooth muscle contraction, migration and proliferation [53]. Moreover, the activation of MLCK by the calcium/calmodulin pathway leads to the regulation of actomyosin cytoskeletal functions, like focal adhesion, stress fibre formation and secretion, ion exchange, cytokinesis, neurite growth cone advancement, cell spreading and endothelial, as well as epithelial, barrier formation [54].

Caveolin 1 (Cav-1) is a membrane protein involved in lipid and membrane traffic, and signal transduction. It is thought to be involved in the interaction between caveolae and cortical actin cytoskeleton, regulating the traveling of caveolae to interior sites [55]. Caveolin 1 is expressed in murine muscle satellite cells and myogenic precursor cells (MPCs), but not in mature muscle fibres. Volonte et al. [56] showed the relevance of Cav-1 in postnatal muscle regeneration, its expression was observed in murine muscle satellite cells and myogenic precursor cells.

Epiregulin (EREG), a potent mitogen secreted by vascular smooth muscle cells, of which it enhances proliferation, was the most downregulated gene [57]. It takes part in vascular remodelling and contributes, as an autocrine and paracrine factor, in the dedifferentiation of vascular smooth muscle cells [58]. Epiregulin is also thought to play a role in the ovulatory process of cycling rats [59].

During long-term in vitro culture, the expression of TGFBR3, which is involved in muscle organ morphogenesis and development and muscle tissue development, was highly downregulated in relation to the initial condition. However, its expression is upregulated during skeletal muscle differentiation [60]. PTGS2, involved in muscle contraction, was observed to be highly downregulated as well. Its low levels of expression may be involved in infertility [61].

## 5. Conclusions

In the current study, the changes in expression of genes belonging to the ontological groups associated with muscle cell morphogenesis, structure, development and differentiation during long-term in vitro culture can be considered as potential markers for the ability of GCs to differentiate and proliferate toward muscle lineage, showing the possibility of their use for muscle regeneration. However, the electron microscopy results revealed heterogenicity among the GCs as well as intracellular structures characteristics of both their physiological state but also stem-like character achieved during culture. Hence, this study serves as a basic molecular reference for the potential further research on the protein level and on the mechanisms underlying GCs toward the muscle lineage. Only the results of such research, together with multiple trials, would allow for better understanding of the possible GC differentiation into muscle-like lineage, as well as potentially to allow to obtain a population of muscle cells that may be used in different clinical situations.

## Figures and Tables

**Table 1:** The sequences of primes used for RT-qPCR analysis.

Gene Name	Abbreviation	Primer Sequences F	Primer Sequences R	Ensembl Accession Number	Product Size (bp)
Oxytocin receptor	OXTR	TTCTTCGTGCAGATGTGGAG	GGACGAGTTGCTCTTTTTGC	ENSG00000111640	234
Keratin 19	KRT19	TTTGAGACGGAACAGGCTCT	AATCCACCTCCACACTGACC	ENSG00000171345	211
Epiregulin	EREG	CCAAGGACGGAAAATGCTTA	AAAATTAGCTGGGCATGGTG	ENSG00000124882	237
Wingless-type MMTV integration site family, member 5A	WNT5A	TGGCTTTGGCCATATTTTTC	CCGATGTACTGCATGTGGTC	ENSG00000114251	199
Transforming growth factor, beta receptor III	TGFBR3	CCAAGATGAATGGCACACAC	CCATCTGGCCAACCACTACT	ENSG00000069702	151
Prostaglandin-enderoperoxide synthase 2	PTGS2	TGAGCATCTACGGTTTGCTG	TGCTTGTCTGGAACAACTGC	ENSG00000073756	158
Caveolin 1	CAV1	TCTCTACACCGTTCCCATCC	CAATCTTGACCACGTCATCG	ENSG00000105974	164
Nexilin	NEXN	AAAAGAAGGCGTTTGCTGAA	CCTCTTCCTCTCCCATTTCC	ENSG00000162614	240
Myosin light chain kinase	MYLK	TTGCTGAGGAAAAGCCTCAT	TTCCCGTCCTCATCGTAGTC	ENSG00000065534	193
Cysteine and glycine-rich protein 3	CSRP3	CCTTGGCACAAGACCTGTTT	TTGTGTAAGGCCTCCAAACC	ENSG00000129170	150
Actin, gamma 2, smooth muscle, enteric	ACTG2	ACCCACAATGTCCCCATCTA	CTCCTTGATGTCTCGCACAA	ENSG00000163017	165
Activating transcription factor 3	ATF3	CGCTGGAATCAGTCACTGTC	AGGCACTCCGTCTTCTCCTT	ENSG00000162772	160
Tropomyosin 1	TPM1	GCTGGTTGAGGAAGAGTTGG	TCGCTCTCAATGATGACCAG	ENSG00000140416	246
SRY (sex determining region Y) -box 9	SOX9	TTGAGCCTTAAAACGGTGCT	CTGGTGTTCTGAGAGGCACA	ENSG00000125398	244
Phosphodiesterase 4b, cAMP-specific	PDE4B	GGAAAAATCCCAGGTTGGTT	AGTGGTGGTGAGGGACTTTG	ENSG00000184588	159
Interleukin 4 receptor	IL4R	CAAGCTCTTGCCCTGTTTTC	TGCACAGAAGCTCCCTTTTT	ENSG00000077238	238
5-hydroxytryptamine (serotonin) receptor 7, adenylate cyclase-coupled	HTR7	GAAGAGTGCTGCCAAACACA	GGTGGCTGCTTTCTGTTCTC	ENSG00000148680	181
Collagen, type VI, alpha 3	COL6A3	ATCTCCTTCATCCCGGACTT	GGACCCATCGATGAGAAAGA	ENSG00000163359	192
Interleukin 1, beta	IL1B	GGGCCTCAAGGAAAAGAATC	TTCTGCTTGAGAGGTGCTGA	ENSG00000125538	205
Beta-actin	ACTB	AAAGACCTGTACGCCAACAC	CTCAGGAGGAGCAATGATCTTG	ENSG00000075624	132
Hypoxanthine-guanine phosphoribosyltransferase	HPRT1	TGGCGTCGTGATTAGTGATG	ACATCTCGAGCAAGACGTTC	ENSG00000165704	141
Glyceraldehyde 3-phosphate dehydrogenase	GAPDH	TCAGCCGCATCTTCTTTTGC	ACGACCAAATCCGTTGACTC	ENSG00000111640	90

**Table 2:** The 10 most significantly upregulated and 10 most significantly downregulated genes involved in muscle cells morphogenesis, structure, development and differentiation.

Gene Symbol	Gene Name	Fold Change	Adj. p. val
**KRT19**	Keratin 19	47.96	0.024
**OXTR**	Oxytocin receptor	38.39	0.001
**CTGF**	Connective tissue growth factor	36.07	0.003
**NEXN**	Nexilin (F actin binding protein)	35.21	0.001
**MYLK**	Myosin light chain kinase	27.45	0.003
**CSRP3**	Cysteine and glycine-rich protein 3 (cardiac LIM protein)	27.41	0.019
**CAV1**	Caveolin 1, caveolae protein, 22kDa	22.41	0.002
**ACTG2**	Actin, gamma 2, smooth muscle, enteric	21.30	0.024
**ATF3**	Activating transcription factor 3	19.20	0.019
**TMP1**	Tropomyosin 1 (alpha)	17.88	<0.001
**SOX9**	SRY (sex determining region Y)-box 9	−5.74	0.020
**WNT5A**	Wingless-type MMTV integration site family, member 5A	−6.31	0.018
**PDE4B**	Phosphodiesterase 4B, cAMP-specific	−7.09	<0.001
**IL4R**	Interleukin 4 receptor	−8.22	0.003
**HTR7**	5-hydroxytryptamine (serotonin) receptor 7, adenylate cyclase-coupled	−8.85	0.005
**COL6A3**	Collagen, type VI, alpha 3	−11.20	0.018
**IL1B**	Interleukin 1, beta	−14.49	0.027
**TGFBR3**	Transforming growth factor, beta receptor III	−20.43	0.021
**PTGS2**	Prostaglandin-endoperioxide synthase 2 (prostaglandin G/H synthase and cyclooxygenase)	−22.65	0.001
**EREG**	Epiregulin	−98.98	<0.001
